# Supplementary material for: RIFINs displayed on malaria-infected erythrocytes bind KIR2DL1 and KIR2DS1
Source: Nature. 2025 Jun 11;643(8074):1363–71. doi: 10.1038/s41586-025-09091-y (PMC12310515; doi:10.1038/s41586-025-09091-y)
Supplement: Supplementary file 1 — This file contains Supplementary Figs. 1 and 2 and Tables 1–3. Supplementary Fig. 1: strategy for expression of RIFINs on iRBCs. Supplementary Fig. 2: gating strategies used for this study. Supplementary Table 1: coverage of rif-lib1 and rif-lib2 for all RIFINs. Supplementary Table 2: summary of mapping results after screening of rif-lib1 and rif-lib2 with KIR2DL1. Supplementary Table 3: oligonucleotides used in the study. [file 41586_2025_9091_MOESM1_ESM.pdf]

nature portfolio

https://doi.org/10.1038/s41586-025-09091-y

Supplementary information

RIFINs displayed on malaria-infected erythrocytes bind KIR2DL1 and KIR2DS1

In the format provided by the authors and unedited

Supplementary Materials

RIFINs displayed on malaria-infected erythrocytes bind KIR2DL1 and KIR2DS1

Akihito Sakoguchi, Samuel G. Chamberlain, Alexander M. Mørch, Marcus Widdess, Thomas E. Harrison, Michael L. Dustin, Hisashi Arase, Matthew K. Higgins, Shiroh Iwanaga

This file includes:

Supplementary Figure 1
Supplementary Figure 2
Supplementary Table 1
Supplementary Table 2
Supplementary Table 3

a

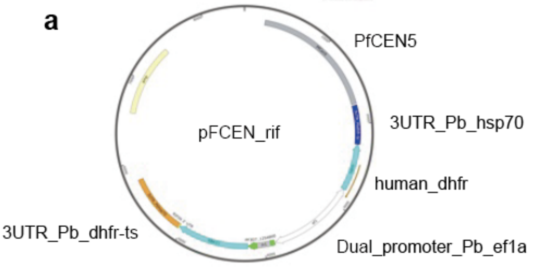

b

atgaaaatccattatattaatatattattgtttcctctaaaattaaatatattgGTAACATTATGTGATAAATAAATAAA
ATATTCATAAATATAATTATTATATCTATATGACTTATATATATAGTTTAAATATACATATTAATTACAAATATATAAAA
TCATAAACTACAAATAAACATTACATATATATGTCACCATTTTTTATAGgtaaatacccacaaaaaaccatccatcacac
cacgtcatatacaaactaccagattattatgcgagtgcgaattatatatgtccaattatgacaacgatcctgaaatgaaa
agggtgatgcaacaatttcatgatcgtacaacacaaaggtttcacgaatacgatgaccgtatgatagaaaaacgccaaaa
atgtaaagatcgatgtaacaaagaaatccaaaaaattattttaaaagacaaaatcgaaaaaaacttagcagaaaaagtgg
aacaaggttgtcttaagtgtgggtgtgggttaggaggtgttgcagcaagtgttggattattcggaggattgggtatctat
agttggaaaagcgtcgcgttggcaaaagctatagctgaaggtgctgctaagggtgctgaggtcggtgaagctgcacgtat
tgcagcagctattgatgcagtcattaaaggaatagaaacaaaatttggtgtatcaactaatggtgttttgggattgaaat
cattatttactgcaaataattataataatgccacaaagattgctggtgccataattgaagaatataaaccatcgtcatgt
ctaacaggtggctctggcgctgataattctatttgcccttgggcgatggaaaatttttttgctgcacggaaaattcctgg
gttcattcaaagggaagctgtttcaatgaatgatgttatagaaaaaactgtaaaaactatcgtctcagatgcccccaaaa
ctgctgaaacagctgcaaaaaaggctactgaagaagctataaaagctagcactgatgcagtagaatctgcatatgctgct
tgtcagactgctattattgcttctgttgttgcattattaattatagttttggttatgatgataatatatttaattttacg
ttatcgtagaaaaaaaaaaaataaagaaaaaactccaatatataaaattattaaaagaatag

Supplementary Figure 1: Strategy for expression of RIFINs on iRBCs

a) The pFCEN-rif plasmid was used for expression of chimeric RIFIN and rifin expression libraries. RIFIN genes were cloned between dual promoter of ef1a and 3'UTR of dhfr-ts gene derived from *P. berghei.* **b)** The DNA sequence of chimeric RBK21 is shown. The green and blue indicate the conserved region of PF3D7_1254800 and the variable region of RBK21, respectively. Black shows the intron sequence of PF3D7_1254800 and is spliced out from mature mRNA of chimeric RBK21.

Supplementary Figure 2: Flow cytometer gating strategy

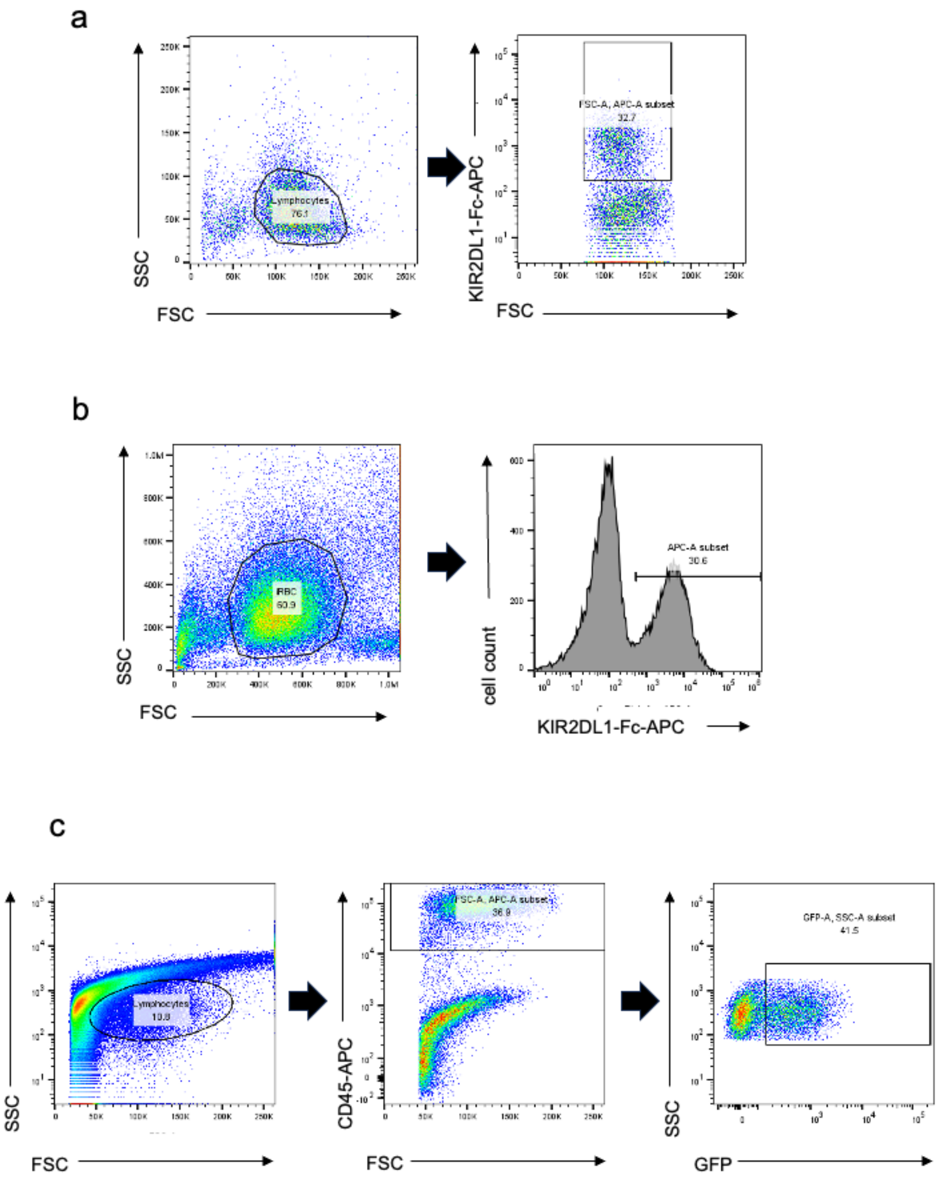

d

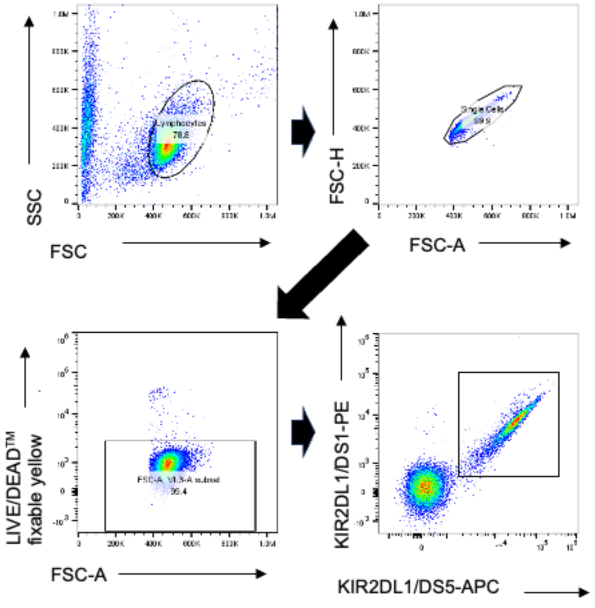
SSC
FSC
Lymphocytes
FSC-H
FSC-A
Single Cells
LIVE/DEAD™
fixable yellow
FSC-A
KIR2DL1/DS1-PE
KIR2DL1/DS5-APC

e

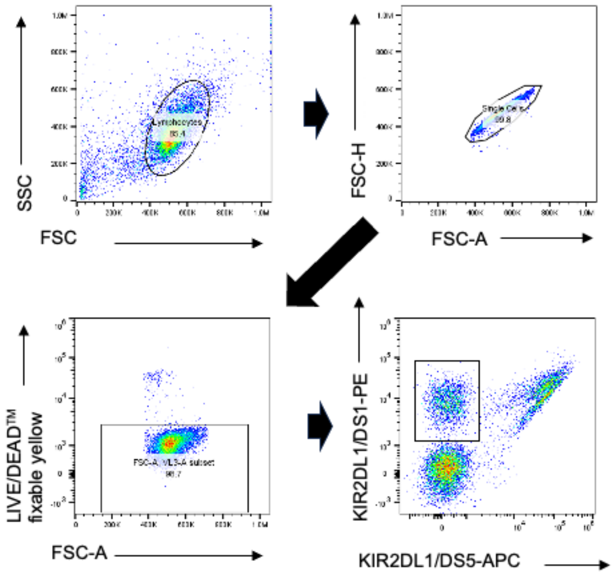
SSC
FSC
Lymphocytes
FSC-H
FSC-A
Single Cells
LIVE/DEAD™
fixable yellow
FSC-A
KIR2DL1/DS1-PE
KIR2DL1/DS5-APC

f

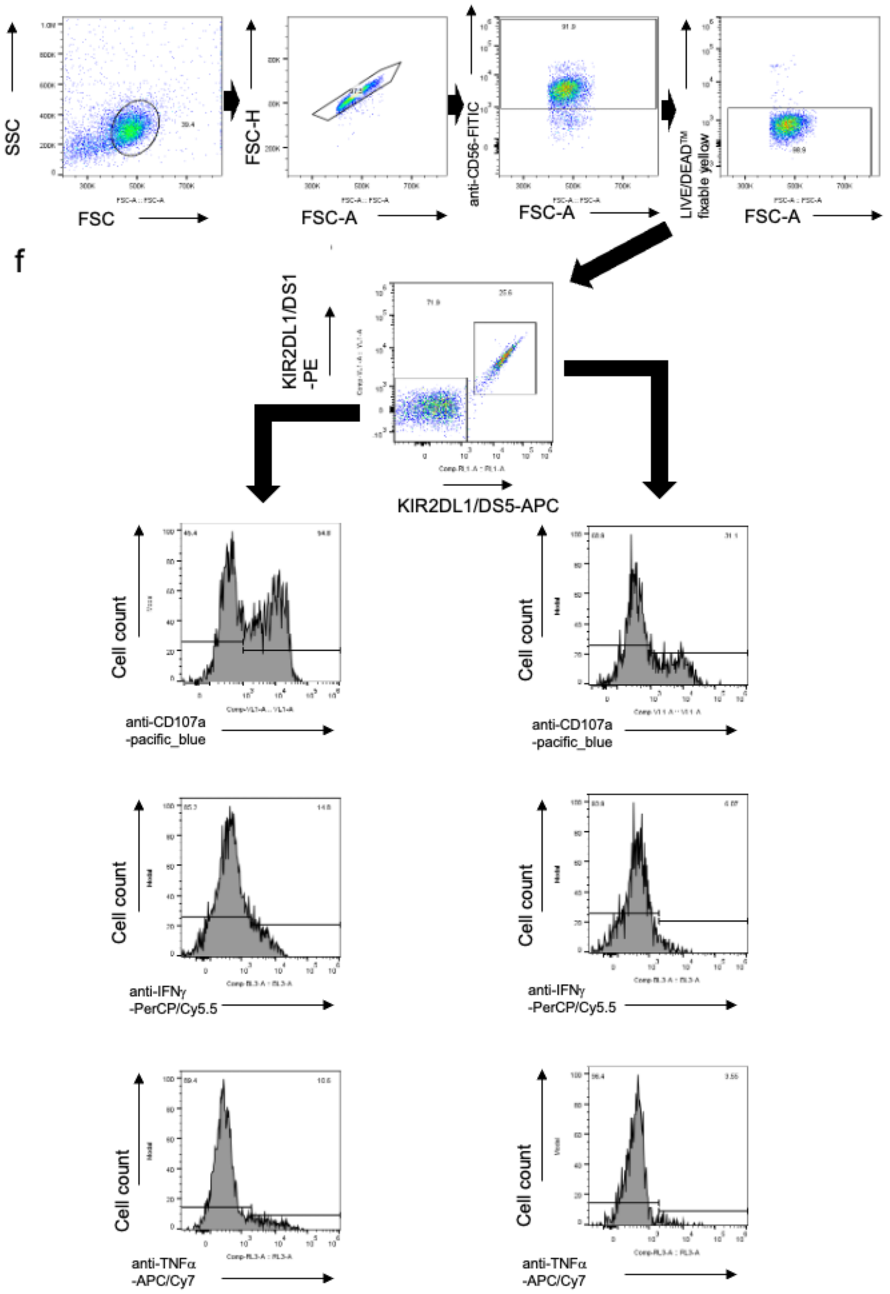
SSC
FSC
FSC-H
FSC-A
anti-CD56-FITIC
FSC-A
LIVE/DEAD™ fixable yellow
FSC-A
KIR2DL1/DS1 -PE
KIR2DL1/DS5-APC
Cell count
anti-CD107a -pacific_blue
Cell count
anti-CD107a -pacific_blue
Cell count
anti-IFNγ -PerCP/Cy5.5
Cell count
anti-IFNγ -PerCP/Cy5.5
Cell count
anti-TNFα -APC/Cy7
Cell count
anti-TNFα -APC/Cy7

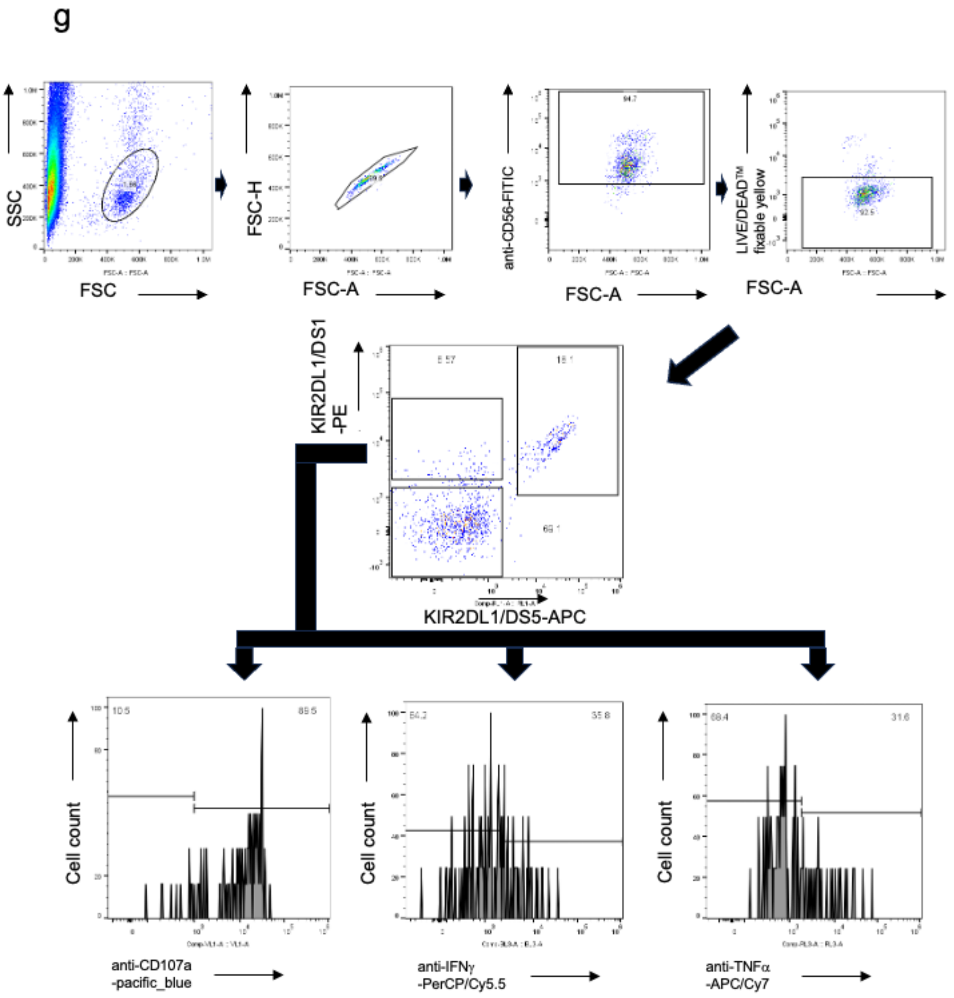

Supplementary Figure 2: Gating strategies used for this study

a) Identification of iRBCs binding to KIR-Fc and KIR2DS1-Fc. This was used for Fig. 1a and Extended Data Fig. 1b. **b)** The binding analysis of transgenic parasites to KIR-Fc fusion proteins. This was used for Fig. 1b and Fig. 4d. **c)** Detection of GFP positive cell in NFAT-reporter cells. This was used for Fig. 3a and Fig. 4e. **d)** Detection of KIR2DL1-positive NK cells in PBMC. This was used for Figure 3b. **e)** Detection of KIR2DS1-positive NK cells in PBMC. This was used for Fig. 4f. **f)** Detection of CD107a positive cells in KIR2DL1-positive NK cells. This was used for Fig. 3c, 3d, 3e, and Extended Data Fig. 6a. **g)** Detection of CD107a positive cells in KIR2DS1-positive NK cells. This was used for Fig. 4g, 4h,4i, and Extended Data Fig. 7b.

Supplementary Table 1: coverage of rif-lib1 and 2 for all RIFINs

gene_id	annotation	mapped-reads_rif_lib1_before	normalization_rif_lib_1_before	mapped-reads_rif_lib2_before	normalization_rif_lib2_before
PF3D7_1000600	rifin	0	0	4	4.7413E-06
PF3D7_0401600	rifin	0	0	0	0
PF3D7_0901100	rifin	0	0	4	4.7413E-06
PF3D7_1253700	rifin	0	0	0	0
PF3D7_0832500	rifin	0	0	0	0
PF3D7_0701800	rifin	0	0	21	2.4892E-05
PF3D7_0700200	rifin	0	0	0	0
PF3D7_0632000	rifin	1	4.9796E-06	5	5.9266E-06
PF3D7_1100400	rifin	1	4.9796E-06	50	5.9266E-05
PF3D7_0732900	rifin	1	4.9796E-06	5	5.9266E-06
PF3D7_0617700	rifin	2	9.9592E-06	8	9.4825E-06
PF3D7_1400400	rifin	3	1.4939E-05	5	5.9266E-06
PF3D7_0600500	rifin	3	1.4939E-05	38	4.5042E-05
PF3D7_0600700	rifin	3	1.4939E-05	349	0.00041368
PF3D7_0100400	rifin	3	1.4939E-05	33	3.9115E-05
PF3D7_1300700	rifin	3	1.4939E-05	17	2.015E-05
PF3D7_1150300	rifin	4	1.9918E-05	12	1.4224E-05
PF3D7_0425900	rifin	4	1.9918E-05	11	1.3038E-05
PF3D7_0413200	rifin	5	2.4898E-05	21	2.4892E-05
PF3D7_1150200	rifin	5	2.4898E-05	35	4.1486E-05
PF3D7_1000400	rifin	5	2.4898E-05	17	2.015E-05
PF3D7_1254400	rifin	6	2.9878E-05	9	1.0668E-05
PF3D7_0300700	rifin	6	2.9878E-05	49	5.808E-05
PF3D7_0223100	rifin	7	3.4857E-05	31	3.6745E-05
PF3D7_1479600	rifin	7	3.4857E-05	9	1.0668E-05
PF3D7_0833000	rifin	7	3.4857E-05	62	7.349E-05
PF3D7_0808800	rifin	8	3.9837E-05	13	1.5409E-05
PF3D7_0100900	rifin	8	3.9837E-05	44	5.2154E-05
PF3D7_0713000	rifin	9	4.4816E-05	205	0.00024299
PF3D7_1479700	rifin	9	4.4816E-05	61	7.2304E-05

PF3D7_0100600	rifin	10	4.9796E-05	71	8.4157E-05
PF3D7_0732500	rifin	11	5.4775E-05	105	0.00012446
PF3D7_0833200	rifin	11	5.4775E-05	78	9.2455E-05
PF3D7_1040500	rifin	12	5.9755E-05	64	7.586E-05
PF3D7_0200700	rifin	12	5.9755E-05	22	2.6077E-05
PF3D7_0324800	rifin	14	6.9714E-05	66	7.8231E-05
PF3D7_0800400	rifin	14	6.9714E-05	40	4.7413E-05
PF3D7_0732400	rifin	15	7.4694E-05	136	0.0001612
PF3D7_0900200	rifin	15	7.4694E-05	242	0.00028685
PF3D7_0732700	rifin	17	8.4653E-05	257	0.00030463
PF3D7_0400900	rifin	17	8.4653E-05	44	5.2154E-05
PF3D7_0300200	rifin	18	8.9633E-05	30	3.5559E-05
PF3D7_0937700	rifin	20	9.9592E-05	44	5.2154E-05
PF3D7_0901500	rifin	20	9.9592E-05	34	4.0301E-05
PF3D7_1101100	rifin	23	0.00011453	178	0.00021099
PF3D7_0937400	rifin	23	0.00011453	135	0.00016002
PF3D7_0400700	rifin	23	0.00011453	77	9.1269E-05
PF3D7_0223400	rifin	24	0.00011951	81	9.6011E-05
PF3D7_1040800	rifin	27	0.00013445	79	9.364E-05
PF3D7_0200600	rifin	29	0.00014441	34	4.0301E-05
PF3D7_1200500	rifin	29	0.00014441	138	0.00016357
PF3D7_1040000	rifin	30	0.00014939	104	0.00012327
PF3D7_1479400	rifin	30	0.00014939	931	0.00110353
PF3D7_0900500	rifin	31	0.00015437	255	0.00030226
PF3D7_1254700	rifin	31	0.00015437	176	0.00020862
PF3D7_0900300	rifin	31	0.00015437	218	0.0002584
PF3D7_1400200	rifin	36	0.00017927	894	0.00105967
PF3D7_1101300	rifin	37	0.00018424	159	0.00018847
PF3D7_0300800	rifin	37	0.00018424	148	0.00017543
PF3D7_1041200	rifin	37	0.00018424	372	0.00044094
PF3D7_0632400	rifin	38	0.00018922	175	0.00020743
PF3D7_0114700	rifin	43	0.00021412	248	0.00029396
PF3D7_1300200	rifin	45	0.00022408	73	8.6528E-05
PF3D7_0632300	rifin	45	0.00022408	226	0.00026788

PF3D7_0833100	rifin	45	0.00022408	139	0.00016476
PF3D7_0401200	rifin	46	0.00022906	364	0.00043145
PF3D7_1100300	rifin	53	0.00026392	134	0.00015883
PF3D7_1300400	rifin	54	0.0002689	95	0.00011261
PF3D7_1400800	rifin	55	0.00027388	181	0.00021454
PF3D7_0937500	rifin	56	0.00027886	433	0.00051324
PF3D7_0400300	rifin	58	0.00028882	169	0.00020032
PF3D7_1255100	rifin	59	0.0002938	128	0.00015172
PF3D7_1041000	rifin	60	0.00029878	107	0.00012683
PF3D7_0632200	rifin	60	0.00029878	474	0.00056184
PF3D7_0632100	rifin	62	0.00030873	229	0.00027144
PF3D7_0900700	rifin	64	0.00031869	76	9.0084E-05
PF3D7_1000500	rifin	68	0.00033861	120	0.00014224
PF3D7_1480000	rifin	70	0.00034857	157	0.00018609
PF3D7_0901300	rifin	71	0.00035355	775	0.00091862
PF3D7_0400500	rifin	75	0.00037347	466	0.00055236
PF3D7_1400500	rifin	76	0.00037845	388	0.0004599
PF3D7_0300500	rifin	76	0.00037845	886	0.00105019
PF3D7_0425700	rifin	78	0.00038841	104	0.00012327
PF3D7_0900400	rifin	81	0.00040335	336	0.00039827
PF3D7_1000200	rifin	82	0.00040833	465	0.00055117
PF3D7_0222600	rifin	83	0.00041331	155	0.00018372
PF3D7_1101200	rifin	84	0.00041829	529	0.00062703
PF3D7_1373100	rifin	86	0.00042824	9219	0.01092743
PF3D7_0800500	rifin	97	0.00048302	117	0.00013868
PF3D7_1254200	rifin	111	0.00055273	190	0.00022521
PF3D7_0222700	rifin	118	0.00058759	249	0.00029514
PF3D7_0413300	rifin	120	0.00059755	485	0.00057488
PF3D7_0632700	rifin	127	0.00063241	190	0.00022521
PF3D7_0701100	rifin	127	0.00063241	742	0.0008795
PF3D7_0200500	rifin	129	0.00064237	180	0.00021336
PF3D7_0101900	rifin	129	0.00064237	292	0.00034611
PF3D7_0101600	rifin	161	0.00080171	244	0.00028922
PF3D7_1372700	rifin	162	0.00080669	86	0.00010194

PF3D7_1372600	rifin	163	0.00081167	256	0.00030344
PF3D7_1254800	rifin	183	0.00091126	571	0.00067682
PF3D7_0808900	rifin	188	0.00093616	323	0.00038286
PF3D7_0115600	rifin	198	0.00098596	582	0.00068985
PF3D7_0732200	rifin	218	0.00108555	970	0.00114976
PF3D7_0201200	rifin	221	0.00110049	634	0.00075149
PF3D7_0937300	rifin	261	0.00129967	878	0.00104071
PF3D7_0100200	rifin	279	0.0013893	1563	0.00185265
PF3D7_0115300	rifin	282	0.00140424	1096	0.00129911
PF3D7_0700500	rifin	292	0.00145404	1039	0.00123154
PF3D7_0100800	rifin	318	0.00158351	1284	0.00152195
PF3D7_0901400	rifin	349	0.00173787	1485	0.00176019
PF3D7_0200200	rifin	376	0.00187232	898	0.00106441
PF3D7_1200200	rifin	411	0.00204661	635	0.00075268
PF3D7_0222500	rifin	419	0.00208645	2717	0.0032205
PF3D7_0401300	rifin	460	0.00229061	159	0.00018847
PF3D7_1040700	rifin	497	0.00247485	1289	0.00152787
PF3D7_1040400	rifin	501	0.00249477	16001	0.01896624
PF3D7_0500400	rifin	531	0.00264416	4331	0.0051336
PF3D7_1040300	rifin	535	0.00266408	2185	0.00258992
PF3D7_1300600	rifin	544	0.00270889	2912	0.00345164
PF3D7_1255000	rifin	587	0.00292302	2795	0.00331296
PF3D7_0600300	rifin	596	0.00296783	1638	0.00194155
PF3D7_1400600	rifin	670	0.00333632	2996	0.00355121
PF3D7_0201000	rifin	712	0.00354546	1416	0.00167841
PF3D7_0115200	rifin	783	0.00389901	11508	0.01364061
PF3D7_0732300	rifin	864	0.00430236	4498	0.00533155
PF3D7_0401400	rifin	960	0.0047804	1829	0.00216794
PF3D7_0421200	rifin	994	0.00494971	518	0.00061399
PF3D7_1041100	rifin	999	0.0049746	1968	0.0023327
PF3D7_0324500	rifin	1220	0.00607509	4075	0.00483016
PF3D7_1373300	rifin	1239	0.0061697	18037	0.02137954
PF3D7_1100500	rifin	1296	0.00645354	5038	0.00597162
PF3D7_1149800	rifin	1296	0.00645354	4249	0.00503641

PF3D7_1300500	rifin	1429	0.00711583	1707	0.00202333
PF3D7_1373000	rifin	1590	0.00791754	3256	0.00385939
PF3D7_0101000	rifin	1652	0.00822627	5022	0.00595266
PF3D7_0631800	rifin	1687	0.00840056	1104	0.00130859
PF3D7_1400300	rifin	2228	0.01109451	14685	0.01740636
PF3D7_0900600	rifin	2303	0.01146798	50923	0.06035984
PF3D7_0833400	rifin	2682	0.01335524	6771	0.00802577
PF3D7_0223200	rifin	2746	0.01367394	11566	0.01370936
PF3D7_1040900	rifin	2768	0.01378349	5403	0.00640426
PF3D7_1479800	rifin	3151	0.01569067	6228	0.00738215
PF3D7_1200300	rifin	3171	0.01579026	40562	0.04807878
PF3D7_0324400	rifin	4012	0.01997809	16002	0.01896742
PF3D7_0700300	rifin	4129	0.0205607	12621	0.01495987
PF3D7_0401000	rifin	4329	0.02155662	13672	0.01620564
PF3D7_1150000	rifin	4504	0.02242805	16359	0.01939058
PF3D7_1373400	rifin	5592	0.02784583	18502	0.02193071
PF3D7_0901000	rifin	6075	0.03025097	74182	0.0879291
PF3D7_1040600	rifin	6133	0.03053979	28582	0.0338787
PF3D7_1254000	rifin	7999	0.03983169	17444	0.02067665
PF3D7_1000300	rifin	8585	0.04274973	35883	0.04253269
PF3D7_1254500	rifin	10859	0.0540733	49887	0.05913185
PF3D7_0532900	rifin	15032	0.0748531	13185	0.01562839
PF3D7_0832800	rifin	16214	0.08073897	58269	0.06906717
PF3D7_1040100	rifin	20181	0.10049298	46124	0.05467151
PF3D7_0500500	rifin	24641	0.12270192	61281	0.07263734

Supplementary Table 2: Summary of mapping results after screening of rif-lib1 and 2 with KIR2DL1

gene_id	annotation	mapped_reads_rif_lib1_K2DL1	normalization_rif_lib1_K2DL1	mapped_reads_rif_lib2_K2DL1	normalization_rif_lib2_K2DL1
PF3D7_0631800	rifin	23993	0.261509788	1692	0.033896268
PF3D7_1040400	rifin	11316	0.123337838	22844	0.457639682
PF3D7_0421200	rifin	9370	0.102127567	756	0.015145141
PF3D7_0223200	rifin	6618	0.072132363	2937	0.058837671
PF3D7_1041100	rifin	4621	0.050366221	1253	0.025101669
PF3D7_1200300	rifin	4562	0.049723155	1120	0.022437246
PF3D7_1400300	rifin	2952	0.032175088	2376	0.047599014
PF3D7_1300600	rifin	2795	0.030463879	1616	0.03237374
PF3D7_1000300	rifin	1647	0.017951345	826	0.016547469
PF3D7_0500500	rifin	1636	0.017831451	799	0.016006571
PF3D7_1150000	rifin	1511	0.016469024	1550	0.031051546
PF3D7_1040100	rifin	1404	0.015302786	259	0.005188613
PF3D7_0324400	rifin	872	0.009504294	494	0.009896428
PF3D7_0115300	rifin	870	0.009482496	202	0.004046718
PF3D7_1254000	rifin	839	0.009144614	308	0.006170243
PF3D7_0324500	rifin	831	0.009057418	822	0.016467336
PF3D7_0222700	rifin	678	0.007389807	51	0.001021696
PF3D7_0532900	rifin	674	0.007346209	210	0.004206984
PF3D7_1255000	rifin	554	0.006038279	308	0.006170243
PF3D7_1479800	rifin	429	0.004675851	86	0.00172286
PF3D7_0901000	rifin	403	0.004392466	952	0.019071659
PF3D7_1040600	rifin	399	0.004348869	93	0.001863093
PF3D7_0115200	rifin	378	0.004119981	29	0.000580964
PF3D7_0632300	rifin	309	0.003367921	436	0.008734499
PF3D7_1254500	rifin	265	0.002888346	392	0.007853036
PF3D7_0401000	rifin	248	0.002703056	148	0.002964922
PF3D7_0700500	rifin	214	0.002332476	48	0.000961596
PF3D7_1101200	rifin	200	0.002179884	76	0.001522527
PF3D7_0100800	rifin	200	0.002179884	122	0.002444057
PF3D7_0832800	rifin	198	0.002158085	392	0.007853036

PF3D7_1149800	rifin	182	0.001983694	53	0.001061763
PF3D7_0413300	rifin	159	0.001733008	155	0.003105155
PF3D7_1373000	rifin	154	0.001678511	2	4.00665E-05
PF3D7_0900500	rifin	152	0.001656712	71	0.001422361
PF3D7_1373400	rifin	149	0.001624014	83	0.00166276
PF3D7_0900600	rifin	145	0.001580416	296	0.005929844
PF3D7_1040700	rifin	127	0.001384226	125	0.002504157
PF3D7_0700300	rifin	125	0.001362428	188	0.003766252
PF3D7_0401400	rifin	123	0.001340629	101	0.002023359
PF3D7_1040900	rifin	115	0.001253433	69	0.001382295
PF3D7_1300500	rifin	106	0.001155339	51	0.001021696
PF3D7_0401300	rifin	103	0.00112264	1	2.00333E-05
PF3D7_0101000	rifin	95	0.001035445	124	0.002484124
PF3D7_1041000	rifin	91	0.000991847	59	0.001181962
PF3D7_0500400	rifin	90	0.000980948	87	0.001742893
PF3D7_1254800	rifin	88	0.000959149	53	0.001061763
PF3D7_1373300	rifin	85	0.000926451	10	0.000200333
PF3D7_0833400	rifin	81	0.000882853	11	0.000220366
PF3D7_0732200	rifin	76	0.000828356	61	0.001222029
PF3D7_0115600	rifin	74	0.000806557	51	0.001021696
PF3D7_0600300	rifin	61	0.000664865	48	0.000961596
PF3D7_0201000	rifin	57	0.000621267	64	0.001282128
PF3D7_0732300	rifin	56	0.000610368	1097	0.021976481
PF3D7_0200200	rifin	55	0.000599468	24	0.000480798
PF3D7_1100500	rifin	54	0.000588569	58	0.001161929
PF3D7_1400600	rifin	50	0.000544971	9	0.000180299
PF3D7_0937300	rifin	46	0.000501373	37	0.00074123
PF3D7_0701100	rifin	45	0.000490474	27	0.000540898
PF3D7_1200200	rifin	45	0.000490474	20	0.000400665
PF3D7_1000200	rifin	40	0.000435977	30	0.000600998
PF3D7_0900700	rifin	39	0.000425077	5	0.000100166
PF3D7_0425700	rifin	34	0.00037058	6	0.0001202
PF3D7_0808900	rifin	33	0.000359681	35	0.000701164
PF3D7_0901400	rifin	32	0.000348781	8	0.000160266

PF3D7_1400800	rifin	31	0.000337882	52	0.001041729
PF3D7_0400900	rifin	27	0.000294284	19	0.000380632
PF3D7_0101900	rifin	26	0.000283385	20	0.000400665
PF3D7_0100200	rifin	25	0.000272486	37	0.00074123
PF3D7_0937400	rifin	21	0.000228888	14	0.000280466
PF3D7_1040300	rifin	20	0.000217988	19	0.000380632
PF3D7_0222500	rifin	19	0.000207089	18	0.000360599
PF3D7_1254200	rifin	18	0.00019619	29	0.000580964
PF3D7_0901300	rifin	16	0.000174391	16	0.000320532
PF3D7_1040800	rifin	13	0.000141692	3	6.00998E-05
PF3D7_1480000	rifin	13	0.000141692	18	0.000360599
PF3D7_0800500	rifin	13	0.000141692	1	2.00333E-05
PF3D7_0223100	rifin	12	0.000130793	1	2.00333E-05
PF3D7_0900300	rifin	12	0.000130793	6	0.0001202
PF3D7_0900400	rifin	12	0.000130793	6	0.0001202
PF3D7_0632700	rifin	11	0.000119894	24	0.000480798
PF3D7_1372600	rifin	11	0.000119894	4	8.0133E-05
PF3D7_0400500	rifin	11	0.000119894	2	4.00665E-05
PF3D7_0632400	rifin	11	0.000119894	2	4.00665E-05
PF3D7_1400200	rifin	11	0.000119894	0	0
PF3D7_0200500	rifin	10	0.000108994	4	8.0133E-05
PF3D7_1041200	rifin	10	0.000108994	1	2.00333E-05
PF3D7_1255100	rifin	10	0.000108994	12	0.000240399
PF3D7_0300200	rifin	8	8.71954E-05	1	2.00333E-05
PF3D7_0201200	rifin	8	8.71954E-05	6	0.0001202
PF3D7_0101600	rifin	8	8.71954E-05	6	0.0001202
PF3D7_0400300	rifin	8	8.71954E-05	11	0.000220366
PF3D7_0632100	rifin	8	8.71954E-05	4	8.0133E-05
PF3D7_0300800	rifin	7	7.62959E-05	3	6.00998E-05
PF3D7_0300500	rifin	7	7.62959E-05	7	0.000140233
PF3D7_0937700	rifin	6	6.53965E-05	10	0.000200333
PF3D7_1101100	rifin	5	5.44971E-05	1	2.00333E-05
PF3D7_0401200	rifin	5	5.44971E-05	4	8.0133E-05
PF3D7_0833000	rifin	5	5.44971E-05	6	0.0001202

PF3D7_1300200	rifin	4	4.35977E-05	0	0
PF3D7_1479400	rifin	4	4.35977E-05	1	2.00333E-05
PF3D7_1000500	rifin	4	4.35977E-05	6	0.0001202
PF3D7_0400700	rifin	4	4.35977E-05	3	6.00998E-05
PF3D7_0833100	rifin	4	4.35977E-05	0	0
PF3D7_0732700	rifin	3	3.26983E-05	1	2.00333E-05
PF3D7_0413200	rifin	3	3.26983E-05	1	2.00333E-05
PF3D7_0223400	rifin	3	3.26983E-05	1	2.00333E-05
PF3D7_1101300	rifin	3	3.26983E-05	1	2.00333E-05
PF3D7_0901500	rifin	3	3.26983E-05	2	4.00665E-05
PF3D7_1150300	rifin	3	3.26983E-05	0	0
PF3D7_0632200	rifin	3	3.26983E-05	5	0.000100166
PF3D7_1100300	rifin	2	2.17988E-05	0	0
PF3D7_1150200	rifin	2	2.17988E-05	0	0
PF3D7_0600500	rifin	2	2.17988E-05	1	2.00333E-05
PF3D7_0937500	rifin	2	2.17988E-05	1	2.00333E-05
PF3D7_0200600	rifin	2	2.17988E-05	0	0
PF3D7_0100600	rifin	2	2.17988E-05	4	8.0133E-05
PF3D7_1200500	rifin	2	2.17988E-05	1	2.00333E-05
PF3D7_0800400	rifin	2	2.17988E-05	4	8.0133E-05
PF3D7_0732500	rifin	1	1.08994E-05	3	6.00998E-05
PF3D7_0732400	rifin	1	1.08994E-05	5	0.000100166
PF3D7_1000600	rifin	1	1.08994E-05	1	2.00333E-05
PF3D7_1254700	rifin	1	1.08994E-05	2	4.00665E-05
PF3D7_1479600	rifin	1	1.08994E-05	0	0
PF3D7_0222600	rifin	1	1.08994E-05	0	0
PF3D7_0808800	rifin	1	1.08994E-05	1	2.00333E-05
PF3D7_0114700	rifin	1	1.08994E-05	0	0
PF3D7_0833200	rifin	1	1.08994E-05	1	2.00333E-05
PF3D7_1000400	rifin	1	1.08994E-05	0	0
PF3D7_1300400	rifin	1	1.08994E-05	0	0
PF3D7_0200700	rifin	1	1.08994E-05	0	0
PF3D7_0100400	rifin	1	1.08994E-05	0	0
PF3D7_0100900	rifin	1	1.08994E-05	1	2.00333E-05

PF3D7_1300700	rifin	1	1.08994E-05	1	2.00333E-05
PF3D7_0324800	rifin	0	0	0	0
PF3D7_1254400	rifin	0	0	0	0
PF3D7_0300700	rifin	0	0	0	0
PF3D7_1040500	rifin	0	0	0	0
PF3D7_0632000	rifin	0	0	1	2.00333E-05
PF3D7_0617700	rifin	0	0	0	0
PF3D7_0401600	rifin	0	0	0	0
PF3D7_0901100	rifin	0	0	0	0
PF3D7_1040000	rifin	0	0	2	4.00665E-05
PF3D7_1373100	rifin	0	0	0	0
PF3D7_1253700	rifin	0	0	0	0
PF3D7_1400500	rifin	0	0	1	2.00333E-05
PF3D7_0832500	rifin	0	0	0	0
PF3D7_0701800	rifin	0	0	0	0
PF3D7_0700200	rifin	0	0	0	0
PF3D7_0713000	rifin	0	0	0	0
PF3D7_1400400	rifin	0	0	0	0
PF3D7_1100400	rifin	0	0	0	0
PF3D7_1479700	rifin	0	0	0	0
PF3D7_0425900	rifin	0	0	0	0
PF3D7_0900200	rifin	0	0	0	0
PF3D7_0600700	rifin	0	0	3	6.00998E-05
PF3D7_1372700	rifin	0	0	0	0
PF3D7_0732900	rifin	0	0	1	2.00333E-05

Supplementary Table 3: Oligonucleotides used in the study.

No	sequence	comments
1	TCCTGAAATGAAAAGGGTRATGSAAAATTTYGATCRWCAAACATCACAAcGWTTTSAACAATAYGAHGA	rif-lib construction
2	TCCTGAAATGAAAAGGGTRATGSAAAATTTYGATCRWCAAACATCACAAcGWTTTSAACAATAYGAHGAA	rif-lib construction
3	GAACAATAATAGATCTYTATTCTTTTAATAATTTTATRTATTGGAGTTTTTTCTTCATTTTTTTTTTTCKWCKATA ACG	rif-lib construction
4	GCAGTCTGACAAGCAGCATATGCAG	5' RACE
5	ATGTCTAACAGGTGGCTCTGGCGC	5' RACE
6	CCTGGGTTCATTCAAAGGGAAGCTGTTTC	5' RACE
7	GCATCAATAGCTGCTGCAATACGTGCAGC	5' RACE
8	ACCTCAGCACCCTTAGCAGCAC	5' RACE
9	CTGCATATGCTGCTTGTCAGACTGC	3' RACE
10	AATGATACGGCGACCACCGAGATCTACACTCTTTCCCTACACGACGCTCTTCCGATCTgcgaattatatatg tccaattatgacaacgatcc	Amplicon-seq
11	CAAGCAGAAGACGGCATACGAGATACATCGGTGACTGGAGTTCAGACGTGTGCTCTTCCGATCTctattc acaaaagaagtacagaacaataatagatc	Amplicon-seq
12	CAAGCAGAAGACGGCATACGAGATACATCGGTGACTGGAGTTCAGACGTGTGCTCTTCCGATCTctattc acaaaagaagtacagaacaataatagatc	Amplicon-seq
13	TAATAACTGCCATTTTTAAAG	Amplicon-seq
14	TATGTTCGTGGCATTCCAC	Amplicon-seq
15	TGGCTTTTGAGTTGAGCTACGGACTCGAGCATGAGGGAGTCCACAGAAAACC	KIR2DL1
16	GTGTGAGTTTTGTCACAAGATTTGGGCTCGAGGTGCAGGTGTCGGGGGTTACC	KIR2DL1
17	TTTTGAGTTGAGCTACGGACTCGAGCATGAGGGAGTCCACAGAAAACCTTCC	KIR2DL2
18	TTTTGTCACAAGATTTGGGCTCGAGGTGCAGGTGTCGGGGGTTACCGGTTTTAG	KIR2DL2
19	TTTTGAGTTGAGCTACGGACTCGAGCATGAGGGAGTCCACAGAAAACC	KIR2DL3
20	TTTTGTCACAAGATTTGGGCTCGAGATGTAGGTGTCTGGGGTTACCGGTTTCG	KIR2DL3
21	TTTTGAGTTGAGCTACGGACTCGAGCATGAGGGTGGTCAGGACAAGCCCTTGC	KIR2DL5
22	TTTTGTCACAAGATTTGGGCTCGAGCAGGTGTCTGCGGATACCAGTTTTGG	KIR2DL5
23	TTTTGAGTTGAGCTACGGACTCGAGGGTCCACACGTGGGTGGTCAGGACAAGCCCTTCC	KIR3DL1
24	TTTTGTCACAAGATTTGGGCTCGAGCAGGTGTCTGGGGTTACCAGATTTGGAGC	KIR3DL1
25	TTTTGAGTTGAGCTACGGACTCGAGCATGAGGGTGGTCAGGACAAGCCCTTGC	KIR3DL2
26	TTTTGTCACAAGATTTGGGCTCGAGCTCGACATGCAGGTGTCTGCAGAT	KIR3DL2
27	TTTTGAGTTGAGCTACGGACTCGAGCAGGACAAGCCCTTCCTCTCTGCC	KIR3DL3
28	TTTTGTCACAAGATTTGGGCTCGAGTCTGGAGTTACCTGTGACAGAAACGGG	KIR3DL3
