# Supplementary material for: RIFINs displayed on malaria-infected erythrocytes bind KIR2DL1 and KIR2DS1
Source: Nature. 2025 Jun 11;643(8074):1363–71. doi: 10.1038/s41586-025-09091-y (PMC12310515; doi:10.1038/s41586-025-09091-y)
Supplement: Supplementary file 2 — Reporting Summary [file 41586_2025_9091_MOESM2_ESM.pdf]

nature portfolio

Corresponding author(s): Matthew K. Higgins and Shiroh Iwanaga

Last updated by author(s): 6-4-25

Reporting Summary

Nature Portfolio wishes to improve the reproducibility of the work that we publish. This form provides structure for consistency and transparency in reporting. For further information on Nature Portfolio policies, see our Editorial Policies and the Editorial Policy Checklist.

Statistics

For all statistical analyses, confirm that the following items are present in the figure legend, table legend, main text, or Methods section.

n/a	Confirmed	
☐	☒	The exact sample size (*n*) for each experimental group/condition, given as a discrete number and unit of measurement
☐	☒	A statement on whether measurements were taken from distinct samples or whether the same sample was measured repeatedly
☐	☒	The statistical test(s) used AND whether they are one- or two-sided<br>*Only common tests should be described solely by name; describe more complex techniques in the Methods section.*
☒	☐	A description of all covariates tested
☒	☐	A description of any assumptions or corrections, such as tests of normality and adjustment for multiple comparisons
☐	☒	A full description of the statistical parameters including central tendency (e.g. means) or other basic estimates (e.g. regression coefficient) AND variation (e.g. standard deviation) or associated estimates of uncertainty (e.g. confidence intervals)
☐	☒	For null hypothesis testing, the test statistic (e.g. *F*, *t*, *r*) with confidence intervals, effect sizes, degrees of freedom and *P* value noted<br>*Give P values as exact values whenever suitable.*
☒	☐	For Bayesian analysis, information on the choice of priors and Markov chain Monte Carlo settings
☒	☐	For hierarchical and complex designs, identification of the appropriate level for tests and full reporting of outcomes
☒	☐	Estimates of effect sizes (e.g. Cohen's *d*, Pearson's *r*), indicating how they were calculated

Our web collection on statistics for biologists contains articles on many of the points above.

Software and code

Policy information about availability of computer code

Data collection	Crystallography data was collected at ESRF ID30-A and Diamond Light Source i03. SPR data were collected using T200 Biacore Software version 2.0 (GE Healthcare). CD data were collected using Spectra Manager Version 2 (Jasco). TIRF microscopy images were acquired using an Olympus cell TIRF-4Line system with a 150x (NA 1.45) oil objective. NGS sequence data of RIFIN expression library were collected using MiSeq (Illumina). Flow cytometry data were collected using Attune NxT (ThermoFisher Scientific). Cell-sorting was performed using SH800 (SONY) or BD LSR II using BD FACSDiva software. In each case, the software is the standard in the field and is available for use by other researchers.
Data analysis	Data analysis was performed as described in the methods section using commercially available or openly accessible software. Software used for crystal data processing are standard and freely available to academic users. Model building and refinement was performed with COOT version ccp4-0.8.9.2 and autoBUSTER v2.10 (Global Phasing Ltd). The SWISS-MODEL webserver was used for homology structure prediction. Chimera v1.16 and ChimeraX v1.5 were used for structure visualisation. GraphPad Prism version 10 was used to generate graphs and for statistical tests. T200 Biacore Evaluation software v1.0 is provided with the Biacore SPR machine and is standard in the field. Microscopy images were analysed using ImageJ (v.1.54b, NIH). Fastq data obtained by screening RIFIN expression library were analyzed using bowtie2 v2.3.4 and featureCounts 2.0.1.

For manuscripts utilizing custom algorithms or software that are central to the research but not yet described in published literature, software must be made available to editors and reviewers. We strongly encourage code deposition in a community repository (e.g. GitHub). See the Nature Portfolio guidelines for submitting code & software for further information.

Data

Policy information about availability of data

All manuscripts must include a data availability statement. This statement should provide the following information, where applicable:

- Accession codes, unique identifiers, or web links for publicly available datasets
- A description of any restrictions on data availability
- For clinical datasets or third party data, please ensure that the statement adheres to our policy

Data within graphs (source data) and uncropped gel and blot images are included with this submission. Crystallographic data is deposited in the protein data bank with accession codes 9F2D and 9HML. Sequence data related to rif-lib1 and -lib2 were deposited at NCBI Gene expression omnibus with accession number GSE286478. All materials are available from the authors.

Human research participants

Policy information about studies involving human research participants and Sex and Gender in Research.

Reporting on sex and gender	N/A
Population characteristics	N/A
Recruitment	N/A
Ethics oversight	N/A

Note that full information on the approval of the study protocol must also be provided in the manuscript.

Field-specific reporting

Please select the one below that is the best fit for your research. If you are not sure, read the appropriate sections before making your selection.

☒ Life sciences ☐ Behavioural & social sciences ☐ Ecological, evolutionary & environmental sciences

For a reference copy of the document with all sections, see nature.com/documents/nr-reporting-summary-flat.pdf

Life sciences study design

All studies must disclose on these points even when the disclosure is negative.

Sample size	Sample sizes are described in figure legends and methods. No statistical method was used to predetermine sample size. Instead, experiments were conducted based on experience of similar studies and statistical significance was assessed on the collected data. Quantitative experiments were typically repeated in technical triplicate. Sample sizes for each experiment were chosen to be consistent with the field norms.
Data exclusions	For CD measurements, data range between 180 and 190 nm were excluded due to high HT values indicating poor quality data in this low UV range.
Replication	The number of repeats for each relevant experiment are given in figure legends and the methods. Typically, experiments were performed in independent technical triplicates. RIFIN-library screening was carried out in biological duplicate.
Randomization	No experiments were randomized and there were no covariants to control. The only assay in which subjectivity is a possible confounder, is in selection of which cells to study to extract the data shown in Figure 3d-f. To avoid this, we acquired fields of cells across the sample based on signals in the IRM channel. All cells within these fields were included in the analysis. This avoided 'cherry-picking' of images, and provided an unbiased assessment. None of the other experiments carried a risk of subjective decisions about data inclusion.
Blinding	The investigators were not blinded to the group allocation during the experiment and/or when assessing the outcome, as analysis were performed on quantitative endpoints that are not subject to investigator bias.

Reporting for specific materials, systems and methods

We require information from authors about some types of materials, experimental systems and methods used in many studies. Here, indicate whether each material, system or method listed is relevant to your study. If you are not sure if a list item applies to your research, read the appropriate section before selecting a response.

Materials & experimental systems

n/a	Involved in the study
☐	☒ Antibodies
☐	☒ Eukaryotic cell lines
☒	☐ Palaeontology and archaeology
☒	☐ Animals and other organisms
☒	☐ Clinical data
☒	☐ Dual use research of concern

Methods

n/a	Involved in the study
☒	☐ ChIP-seq
☐	☒ Flow cytometry
☒	☐ MRI-based neuroimaging

Antibodies

Antibodies used	anti-KIR2DL1/DS1 (Milteny Biotec, 130-118-973, 1:100 dilution)<br>anti-KIR2DL1/DS5 (R&D system, MAB1844-SP, 1:100 dilution)<br>anti-FLAG antibody (Sigma-Aldrich, F1804, 1:200 dilution)<br>APC-conjugated anti-human IgG Fc antibody (Jackson ImmunoResearch,109-136-098, 1:100 dilution)<br>FITC-conjugated CD56 (Biolegend, 318303, 1:100 dilution),<br>PacificBlue-conjugated anti-human CD107a (Biolegend, 328623, 1:100 dilution)<br>PerCP/Cy5.5-conjugated anti-human IFN-g (Biolegend, 506527, 1:100 dilution),<br>APC/Cy7-conjugated anti-human TNF-a (Biolegend, 502943, 1:100 dilution),<br>APC-conjugated anti-mouse-CD45 (Biolegend, 103111, 1:100 dilution)<br>anti-KIR3DL2 antibody (Biolegend, 389602, 1:100 dilution)<br>anti-KIR3DL2 (Biolegend, 389602, 1:100 dilution)<br>anti-KIR3DL3 (R&D Systems, FAB8919P, 1:100 dilution).<br>FITC-conjugated anti-KIR2DL1/DL5 (R&D Systems, FAB1844F, 1:100 dilution)<br>PE-conjugate anti-KIR2DL2/DL3/DS2 (Biolegend, 312605, 1:100 dilution),<br>PE-conjugated anti-KIR2DL5 (Miltenyi Biotec, 130-096-199, 1:100 dilution),<br>FITC-conjugated anti-KIR3DL1 (Biolegend, 312705, 1:100 dilution)
Validation	The antibodies used in this study were obtained from commercial vendors. We selected commercially available antibodies based on the validation provided by the manufacturers for their use in flow cytometry. Detailed validation information can be accessed on the manufacturers' websites using the details provided in the "Antibodies Used" section above.

Eukaryotic cell lines

Policy information about cell lines and Sex and Gender in Research

Cell line source(s)	Commercial FreestyleTM 293 and Expi 293F GNTI-TM cells were purchased from Thermo Fisher.<br>HEK293T was obtained from RIKEN cell Bank.<br>NKL were generously gifted by L.L. Lanier at the University of California San Francisco.<br>The human erythroleukemia cell line, K562, was obtained from the Cell Resource Centre for Biomedical Research, Institute of Development, Ageing and Cancer, Tohoku University.<br>Plasmodium falciparum 3D7 (ID: MRA845) was obtained from BEI Resources.
Authentication	FreestyleTM 293 and Expi 293F GNTI-TM cells were authenticated by Thermo Fisher. Example of citation; PMID:14701821.<br>HEK293T cell was authenticated by RIKEN cell Bank. Example of citation: PMID: 39772386.<br>NKL was described in Exp. Hematol. 1996 Feb;24(3):406-15. PMID: 8599969<br>K562, was authenticated by the Cell Resource Centre for Biomedical Research, Institute of Development, Ageing and Cancer, Tohoku University. Example of citation: PMID: 29186116<br>Plasmodium falciparum 3D7 strain was authenticated by BEI Resources.
Mycoplasma contamination	Each cell line listed above was regularly tested by mycoplasma contamination by PCR.
Commonly misidentified lines<br>(See ICLAC register)	Plasmodium falciparum 3D7 is not listed in ICLAC.

Flow Cytometry

Plots

Confirm that:

☒ The axis labels state the marker and fluorochrome used (e.g. CD4-FITC).

☒ The axis scales are clearly visible. Include numbers along axes only for bottom left plot of group (a 'group' is an analysis of identical markers).

☒ All plots are contour plots with outliers or pseudocolor plots.

☒ A numerical value for number of cells or percentage (with statistics) is provided.

Methodology

Sample preparation	The iRBCs at schizont stage were obtained by 70% - 40% percoll density gradient centrifugation. Human peripheral blood mono-nucleated cells (PBMC) of healthy donors were isolated from fresh blood samples by Ficoll-Paque (Leucosep.
Instrument	Sample were analyzed using Attune NxT (ThermoFisher Scientific). The iRBCs , on which expressed KIR2DL1-binding RIFINs, were sorted using SH800 (SONY). KIR-Fc validation assay was analysed using BD LSR II.
Software	Data collection: Attune NxT software (for Attune NxT), SH800s software (for SH800), BD FACSDiva (for BD LSR II)<br>Data analysisi: FlowJo v10.10.0
Cell population abundance	The sorted iRBC, which was infected with field-isoalated parasites, were > 32.7 % KIR2DL-1 positive (FIgure 1a). The iRBCs expressing KIR2DL1-binding RIFINs were sorted from RIFIN expression libraries and were 2.13- 1.63 % were positive in the iRBC population of libraries (Figure 1d).
Gating strategy	All gating strategy are provided in Extended data Figure 12

☒ Tick this box to confirm that a figure exemplifying the gating strategy is provided in the Supplementary Information.
